# Genetic Deletion of Afadin Causes Hydrocephalus by Destruction of Adherens Junctions in Radial Glial and Ependymal Cells in the Midbrain

**DOI:** 10.1371/journal.pone.0080356

**Published:** 2013-11-13

**Authors:** Hideaki Yamamoto, Tomohiko Maruo, Takashi Majima, Hiroyoshi Ishizaki, Miki Tanaka-Okamoto, Jun Miyoshi, Kenji Mandai, Yoshimi Takai

**Affiliations:** 1 Division of Pathogenetic Signaling, Department of Biochemistry and Molecular Biology, Kobe University Graduate School of Medicine, Kobe, Hyogo, Japan; 2 Division of Molecular and Cellular Biology, Department of Biochemistry and Molecular Biology, Kobe University Graduate School of Medicine, Kobe, Hyogo, Japan; 3 CREST, Japan Science and Technology Agency, Kawaguchi, Saitama, Japan; 4 Department of Molecular Biology and Biochemistry, Osaka University Graduate School of Medicine, Suita, Osaka, Japan; 5 Department of Molecular Biology, Osaka Medical Center for Cancer and Cardiovascular Disease, Osaka, Japan; Rutgers University -New Jersey Medical School, United States of America

## Abstract

Adherens junctions (AJs) play a role in mechanically connecting adjacent cells to maintain tissue structure, particularly in epithelial cells. The major cell–cell adhesion molecules at AJs are cadherins and nectins. Afadin binds to both nectins and α-catenin and recruits the cadherin-β-catenin complex to the nectin-based cell–cell adhesion site to form AJs. To explore the role of afadin in radial glial and ependymal cells in the brain, we generated mice carrying a nestin-Cre-mediated conditional knockout (cKO) of the *afadin* gene. Newborn *afadin*-cKO mice developed hydrocephalus and died neonatally. The *afadin*-cKO brain displayed enlarged lateral ventricles and cerebral aqueduct, resulting from stenosis of the caudal end of the cerebral aqueduct and obliteration of the ventral part of the third ventricle. Afadin deficiency further caused the loss of ependymal cells from the ventricular and aqueductal surfaces. During development, radial glial cells, which terminally differentiate into ependymal cells, scattered from the ventricular zone and were replaced by neurons that eventually covered the ventricular and aqueductal surfaces of the *afadin*-cKO midbrain. Moreover, the denuded ependymal cells were only occasionally observed in the third ventricle and the cerebral aqueduct of the *afadin*-cKO midbrain. Afadin was co-localized with nectin-1 and N-cadherin at AJs of radial glial and ependymal cells in the control midbrain, but these proteins were not concentrated at AJs in the *afadin*-cKO midbrain. Thus, the defects in the *afadin*-cKO midbrain most likely resulted from the destruction of AJs, because AJs in the midbrain were already established before *afadin* was genetically deleted. These results indicate that afadin is essential for the maintenance of AJs in radial glial and ependymal cells in the midbrain and is required for normal morphogenesis of the cerebral aqueduct and ventral third ventricle in the midbrain.

## Introduction

The aqueductal and ventricular walls of the brain are covered by a succession of epithelial cells, progressing from neuroepithelial to radial glial and eventually ependymal cells, over the course of development [1], [2]. These epithelial cells attach to each other via a junctional complex of adherens junctions (AJs) and tight junctions (TJs) to form a monolayer sheet. AJs play a role in mechanically connecting adjacent cells to maintain tissue structure particularly in many types of epithelial and endothelial cells, including neuroepithelial, radial glial, and ependymal cells [3]. TJs are developed in addition to AJs in epithelial and endothelial cells, including neuroepithelial and ependymal cells, but not in radial glial cells [2], [4]. TJs are localized at the most apical side of the cell–cell adhesion site and AJs are formed just at the basal side of TJs in epithelial cells [5]. TJs are crucial for the barrier function that prevents the passage of soluble molecules through the gaps between cells [4]. The formation and maintenance of TJs are regulated by AJs [6].

The major cell adhesion molecules (CAMs) at AJs are cadherins and nectins [7], [8]. Cadherins are key Ca^2+^-dependent CAMs with a single transmembrane segment and comprise a family consisting of over 100 members [8]. Cadherins directly bind to the adherens junctional components β-catenin and p120^ctn^ [9]. β-Catenin in turn interacts with α-catenin, which binds many peripheral membrane proteins, such as vinculin, α-actinin, and EPLIN [9], [10], while p120^ctn^ binds PLEKHA7 [11]. Nectins are Ca^2+^-independent immunoglobulin (Ig)-like CAMs with a single transmembrane segment and comprise a family consisting of four members: nectin-1, -2, -3, and -4 [7]. Nectins first form cell–cell adhesion and then recruit cadherins to the nectin-based cell–cell adhesion site to form AJs [7]. Nectins and cadherins are further involved in the formation of TJs [12] of which major CAMs are junction adhesion molecules, occludin, and claudins [13].

Afadin is localized at AJs in epithelial and endothelial cells and regulates the formation of AJs in cooperation with nectins and cadherins [14]. Afadin is an actin filament-binding protein, encoded by the *MLLT4/AF-6* gene [15]. Afadin has some splicing isoforms and the longest one, l-afadin, hereafter referred to as afadin, is ubiquitously expressed including epithelial cells. Afadin was originally isolated as an actin filament-binding protein of which the nucleotide sequence was similar to that of the *AF6* gene [15]. The *AF-6* gene was originally reported as a fusion partner of the *MLL* (alias, *ALL-1*) gene in pediatric acute myeloid leukemia with chromosome translocation [16]. Afadin contains multiple domains and interacts with many proteins including CAMs and their associating molecules and signaling molecules [14]. Afadin binds to not only nectins but also α-catenin, p120^ctn^, and PLEKHA7 and recruits the cadherin-β-catenin complex to the nectin-based cell–cell adhesion site to form AJs [7], [14], [17]–[20]. In addition, afadin binds to ZO-1 and recruits junctional adhesion molecules, occludin, and claudins to the apical side of the nectin-based cell–cell adhesion sites to form TJs [12], [21]–[25].

We previously reported that *afadin* knockout (KO) mice showed developmental defects in stages during and after gastrulation and that these mice died during early embryonic stages [26], hence making it difficult to analyze the effects of the afadin deficiency on the brain. To assess the function of afadin in the brain, we crossed *afadin*-floxed mice with nestin-Cre transgenic mice [27], [28] and generated the central nervous system (CNS)-specific conditional KO (cKO) mice of the *afadin* gene. The *nestin* gene encodes an intermediate filament protein strongly expressed in neural progenitor cells of CNS tissues from around embryonic day (E) 10.5 [28]. We found here that the *afadin*-cKO mice developed hydrocephalus.

Hydrocephalus is the pathological condition with an excessive accumulation of cerebrospinal fluid (CSF) in the ventricle and caused by overproduction of CSF by the choroid plexus, a defect of CSF reabsorption, and/or impaired CSF flow [29]. CSF flows from the lateral ventricles through the paired foramina of Monro into the third ventricle, then passes the cerebral aqueduct and finally reaches the fourth ventricle. Congenital hydrocephalus is a developmental brain disorder. In humans, its incidence is about 1–3 in every 1,000 live birth. The cerebral aqueduct is the most common site of intraventricular blockage of the CSF [30]. Evidence has accumulated that neuroepithelial and ependymal cells lining the ventricular and aqueductal walls of the developing brain play a key role in the onset and evolution of congenital hydrocephalus [31].

In this study, we aimed to clarify the role of afadin in the brain by using the nestin-Cre-mediated cKO mice of the *afadin* gene and described that the *afadin*-cKO mice developed noncommunicative hydrocephalus, owing to the developmental defect in the midbrain.

## Results

### Hydrocephalus in nestin-Cre-mediated *afadin*-cKO mice

**Figure 1:**
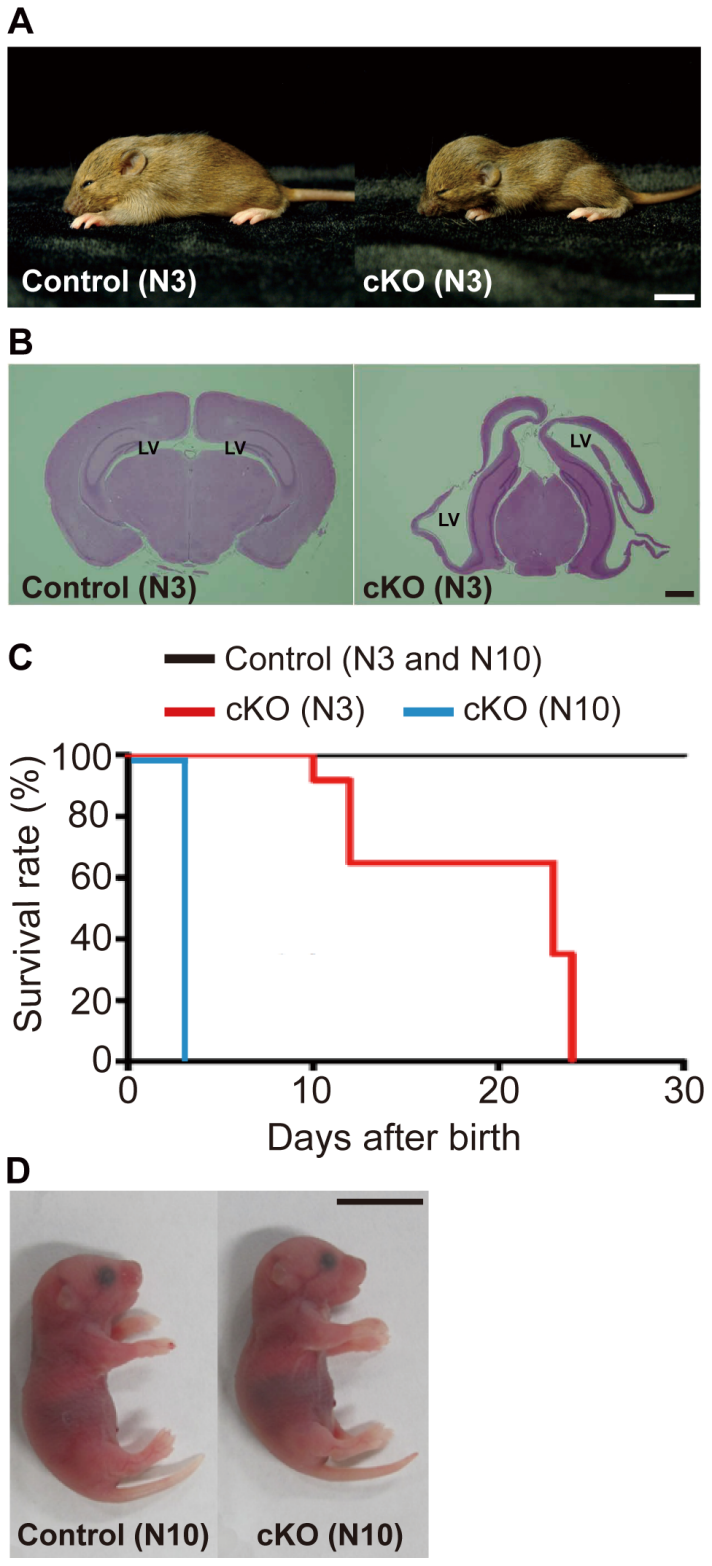
Gross phenotypes of the nestin-Cre-mediated cKO mice of the *afadin* gene. (**A**) Appearance of the control and *afadin*-cKO mice at P14 from the third backcross (N3) generation. (**left panel**) The control mouse; and (**right panel**) the *afadin*-cKO mouse. Scale bar: 1 cm. (**B**) Brain sections stained with HE in the control and *afadin*-cKO mice at P14 from the N3 generation. (**left panel**) The control mouse; and (**right panel**) the *afadin*-cKO mouse. LV, lateral ventricle. Scale bar: 1 mm. (**C**) Kaplan–Meier survival curves of the N3 generation of the control (n = 39) and *afadin*-cKO (n = 13) mice and the N10 generation of the control (n = 10) and *afadin*-cKO (n = 12) mice. (**D**) Appearance of the control and *afadin*-cKO mice at P0 from the N10 generation. (**left panel**) The control mouse; and (**right panel**) the *afadin*-cKO mouse. Scale bar: 1 cm.

We generated the nestin-Cre-mediated cKO mice of the *afadin* gene as described in Materials and Methods. When the *afadin*-cKO mice were backcrossed into the genetic background of C57/BL6 mice from the mixed 129/Sv-C57/BL6 background, the *afadin*-cKO mice at postnatal day (P) 14 from the third backcross (N3) generation displayed the dome-shaped head and enlarged ventricles (**Fig. 1, A and B**) and died by P21 (**Fig. 1C**). These results indicate that the mixed 129/Sv-C57/BL6 background of the *afadin*-cKO mice developed hydrocephalus.

**Figure 2:**
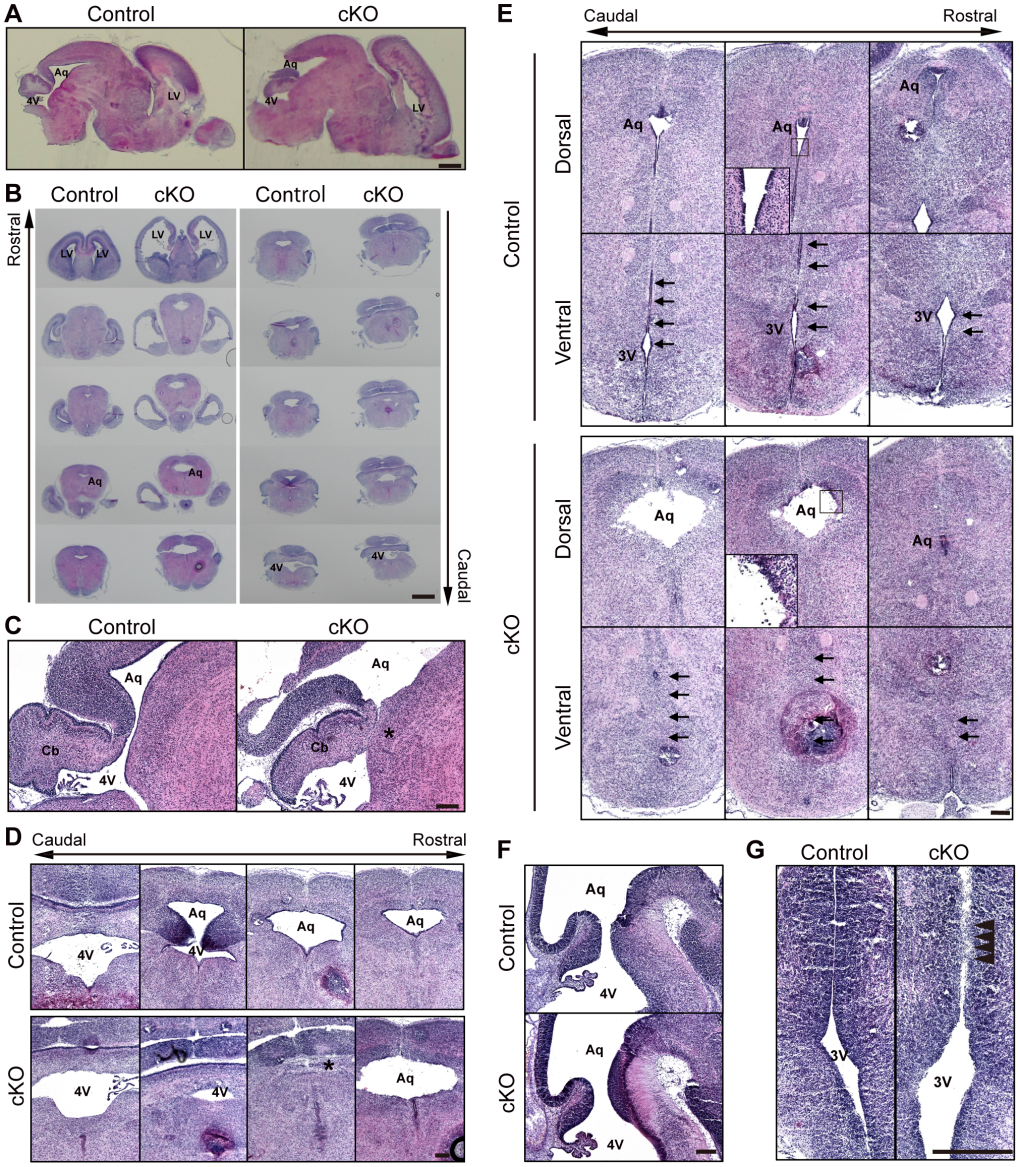
Histological phenotypes of the *afadin*-cKO brain. Sections were stained by HE. (**A**) the sagittal sections of the whole brain; (**B**) the coronal sections of the whole brain; (**C**) the sagittal sections of the fourth ventricle and the cerebral aqueduct; (**D**) the coronal sections of the fourth ventricle and the cerebral aqueduct; (**E**) the coronal sections of the cerebral aqueduct and the third ventricle; (**F**) the sagittal sections of the cerebral aqueduct; and (**G**) the coronal sections of the third ventricle in the control and *afadin*-cKO mice at P0 (**A–E**) and E13.5 (**F–G**). Asterisks: stenosed cerebral aqueduct. Arrows: ventral part of the third ventricle. Arrowheads: obliterated third ventricle. LV, lateral ventricle; Aq, aqueduct; 4V, fourth ventricle; 3V, third ventricle; Cb, cerebellum primordium. Scale bars: 1 mm (**A and B**) and 200 µm (**C–G**).

It was previously shown that the C57/BL6 background mice developed more severe hydrocephalus than the mixed 129/Sv-C57/BL6 background mice [32]–[37]. Consistently, when the *afadin*-cKO mice were backcrossed into the genetic background of C57/BL6 mice, the congenic offspring (N10) were born in the expected Mendelian ratio, but all of the *afadin*-cKO mice died within 3 days after birth (**Fig. 1C**). The appearance of the whole body of the *afadin*-cKO mice at P0 was indistinguishable from that of the control mice (**Fig. 1D**). However, in hematoxylin and eosin (HE) staining of brain sections at P0, they displayed obviously enlarged lateral ventricles and the cerebral aqueduct as compared with those of the control brain (**Fig. 2, A–E**), while the forth ventricle of the *afadin*-cKO mice was similar to that of the control mice (**Fig. 2, A–D**). The cerebral cortex of the *afadin*-cKO mice was apparently thinner than that of the control mice (**Fig. 2, A and B**). These gross phenotypes suggest that the C57/BL6 background *afadin*-cKO mice show developmental defects with severe hydrocephalus.

### Stenosis of the caudal end of the cerebral aqueduct and obliteration of the ventral part of the third ventricle in the *afadin*-cKO brain

To determine the defect responsible for hydrocephalus in the *afadin*-cKO mice, we first examined whether stenosis and/or obliteration are observed in the *afadin*-cKO brain. In histological analysis, the caudal end of the cerebral aqueduct, which connects the third ventricle to the fourth ventricle, was stenosed at P0 (**Fig. 2** **, C and D, asterisks**). Furthermore, the ventral part of the third ventricle was obliterated in the *afadin*-cKO mice at P0 (**Fig. 2E** **, arrows**). The dorsal and ventral tissues beneath the stenosed area of the cerebral aqueduct and the bilateral tissues in the obliterated area of the third ventricle were fused. In contrast, the cerebral aqueduct of the *afadin*-cKO brain at E13.5 was almost normal and was indistinguishable from that of the control brain (**Fig. 2F** **, arrowheads**), while the ventral part of the third ventricle of the *afadin*-cKO brain was partly obliterated (**Fig. 2G**). These results indicate that the genetic deletion of *afadin* causes stenosis of the caudal end of the cerebral aqueduct and obliteration of the ventral part of the third ventricle, leading to the onset and progression of hydrocephalus.

### Disappearance of ependymal cells from the surface of the caudal ventral part of the third ventricle and the rostral cerebral aqueduct of the *afadin*-cKO brain

**Figure 3:**
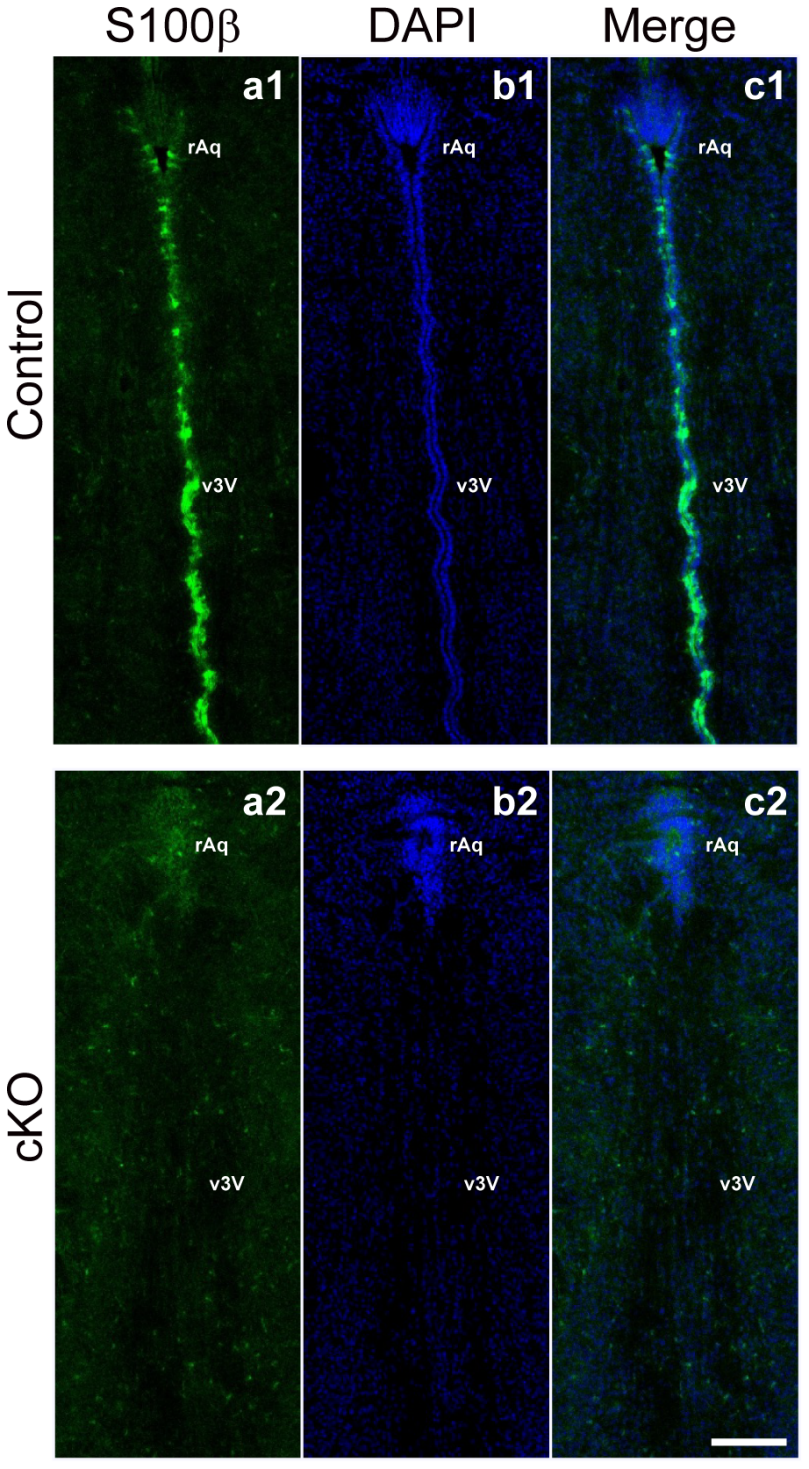
Ependymal cells of the third ventricle and the cerebral aqueduct in the *afadin*-cKO midbrain. The coronal sections were stained with the anti-S100β Ab and DAPI in the control and *afadin*-cKO midbrains at P0; (**a1 and a2**) with the anti-S100β Ab; and (**b1 and b2**) with DAPI. (**a1–c1**) the control midbrain; and (**a2–c2**) the *afadin*-cKO midbrain. rAq, rostral aqueduct; v3V, ventral part of the third ventricle. Scale bar: 100 µm.

It was shown that stenosis of the cerebral aqueduct is caused by denudation of ependymal cells and appearance of reactive astrocytes [31], [38]. We therefore examined whether these phenotypes were observed in the *afadin*-cKO brain. Indeed, cells were occasionally observed in the cerebral aqueduct and the third ventricle near the walls of the *afadin*-cKO mice (**Fig. 2E**, insets, and 2G, arrowheads****), suggesting that these cells might be denuded ependymal cells. Ependymal cells mature first in the narrow regions, such as the caudal ventral part of the third ventricle and the rostral cerebral aqueduct till the birth [39] and mature ependymal cells can be identified by the expression of the S100β protein [40]. In the control brain at P0, the intense immunofluorescence signal for the S100β protein was observed in the cells covering the surface of the caudal ventral part of the third ventricle and the rostral cerebral aqueduct (**Fig. 3**). In contrast, the signal for the S100β protein was not observed at the rostral aqueduct or the obliterated ventral part of the third ventricle in the *afadin*-cKO brain, suggesting the disappearance of ependymal cells in the *afadin*-cKO brain (**Fig. 3**). Conversely, the signal for glial fibrillary acidic protein, a marker for reactive astrocytes [41], was not observed in the cerebral aqueduct or the third ventricle of the control and the *afadin*-cKO mice at P0 (data not shown), indicating that astrocytes were not activated in the *afadin*-cKO brain. These results indicate that the genetic deletion of *afadin* causes the disappearance of ependymal cells at the caudal ventral part of the third ventricle and the rostral cerebral aqueduct, but does not activate the astrocytes.

### Aberrant location of radial glial cells and neurons in the *afadin*-cKO midbrain

**Figure 4:**
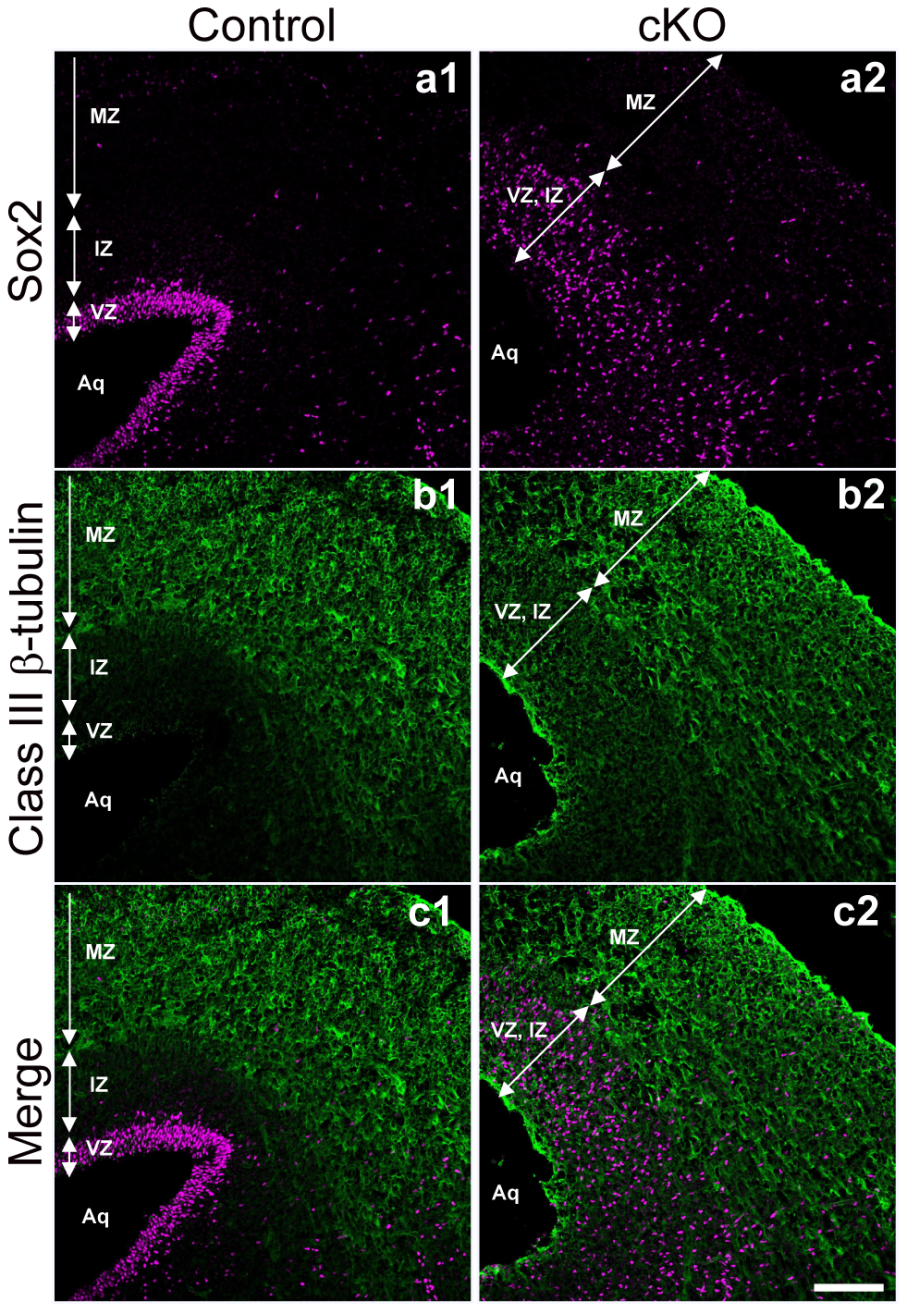
Distribution of radial glial cells and neurons in the *afadin*-cKO midbrain. The coronal sections were stained with the anti-Sox2 and anti-class III β-tubulin Abs in the control and *afadin*-cKO midbrains at E16.5; (**a1 and a2**) with the anti-Sox2 Ab; and (**b1 and b2**) with the anti-class III β-tubulin Ab. (**a1–c1**) The control midbrain; and (**a2–c2**) the *afadin*-cKO midbrain. VZ, ventricular zone; IZ, intermediate zone; MZ, mantle zone; Aq, aqueduct. Scale bar: 100 µm.

Because ependymal cells are produced from radial glial cells [42], we examined whether the genetic deletion of *afadin* affected radial glial cells in the midbrain. We performed immunostaining with an anti-Sox2 antibody (Ab) to label radial glial cells in the midbrain at E16.5. Sox2-positive cells, which were normally restricted in the ventricular zone, were scattered throughout the ventricular and intermediate zones in the *afadin*-cKO midbrain (**Fig. 4** **, a1 and a2**). Moreover, we stained the control and *afadin*-cKO midbrains with an anti-class III β-tubulin Ab to label neurons [43]. In the control midbrain, the immunofluorescence signal was detected in the mantle zone, and the weak signal was observed in the intermediate zone (**Fig. 4** **, b1 and b2**). Conversely, in the *afadin*-cKO midbrain, the intense signal was observed at the surface of the cerebral aqueduct of the midbrain, in addition to the signal observed in the ventricular, intermediate, and mantle zones. These results indicate that radial glial cells were mislocalized in the *afadin*-cKO midbrain and that the surface of the cerebral aqueduct of the *afadin*-cKO midbrain was covered by neurons instead of radial glial and/or ependymal cells.

### Localization of afadin at AJs of radial glial and ependymal cells at the cerebral aqueduct and the third ventricle

**Figure 5:**
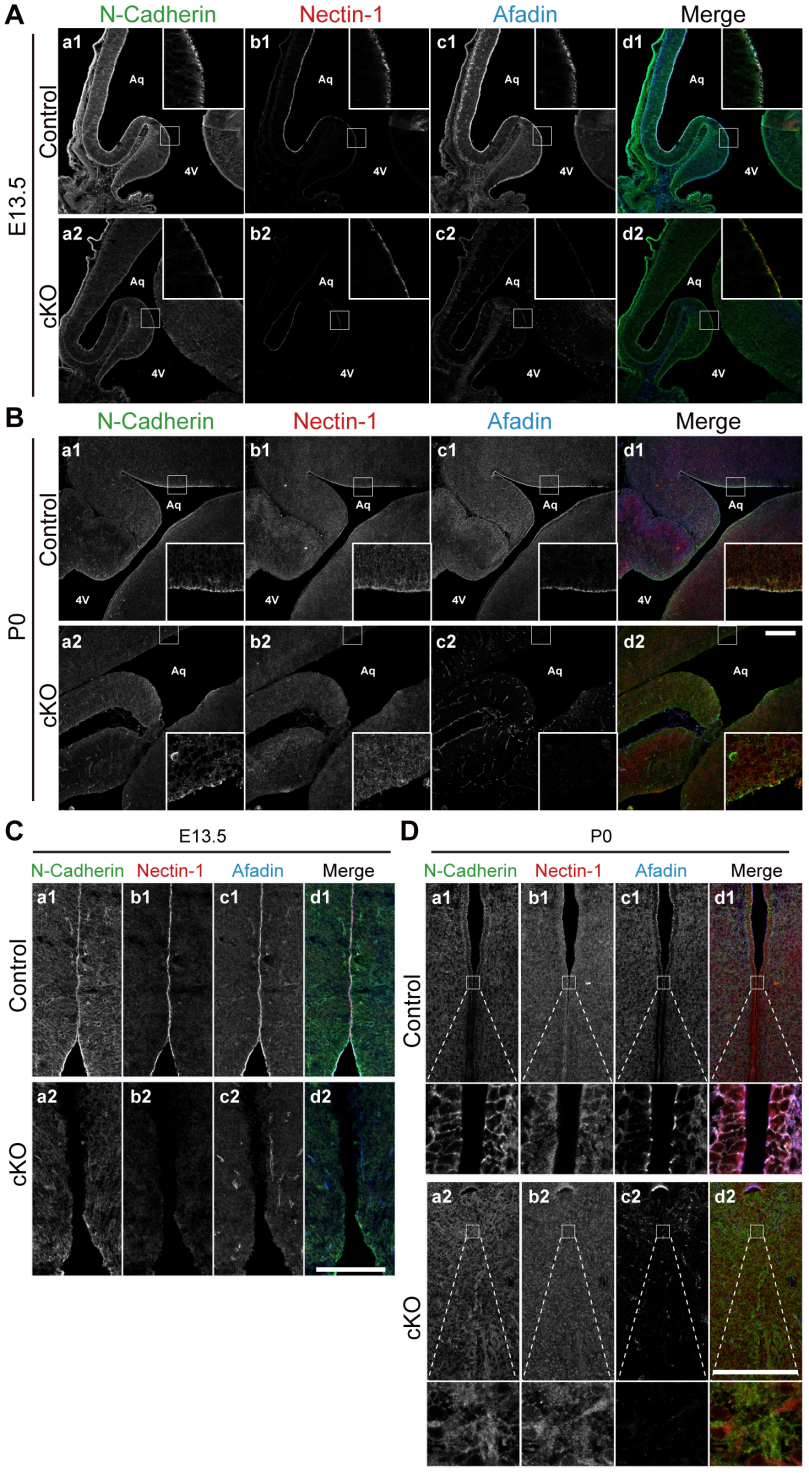
Localization of afadin and AJ proteins in the *afadin*-cKO cerebral aqueduct and third ventricle. The sections of the cerebral aqueduct and the third ventricle were stained with the indicated Abs in the control and *afadin*-cKO mice at E13.5 and P0. (**A and B**) The cerebral aqueduct; (**C and D**) the third ventricle; (**A and C**) at E13.5; and (**B and D**) at P0; (**a1 and a2**) with the anti-N-cadherin Ab; (**b1 and b2**) with the anti-nectin-1 Ab; and (**c1 and c2**) with the anti-afadin Ab. (**a1–d1**) The control mice; and (**a2–d2**) the *afadin*-cKO mice. Aq, aqueduct; 4 V, fourth ventricle. Scale bars: 200 µm.

Next, we examined the localization of afadin at the cerebral aqueduct and the third ventricle with immunofluorescence microscopy using the Ab that specifically recognizes l-afadin, but not s-afadin (l-afadin Ab). The immunofluorescence signal for afadin was highly concentrated at the apical surface of the cerebral aqueduct and the third ventricle at E13.5 and P0 in the control mice (**Fig. 5** **, A–D, c1**). The signal for afadin was co-localized with those for nectin-1 and N-cadherin, although the signal for N-cadherin was more broadly distributed along the lateral plasma membrane (**Fig. 5** **, A–D, a1–d1**). Because the apical surface of the lateral ventricles is covered by radial glial cells until around E15 and then covered by ependymal cells that are produced from radial glial cells between E14 and E16 [42], and N-cadherin and nectin-1 are major CAMs at AJs in the nervous tissues [44], [45], these results indicate that afadin is localized at AJs in radial glial and ependymal cells at the cerebral aqueduct and the third ventricle.

### Gradual deletion of *afadin* and destruction of AJs in the *afadin*-cKO embryonic brain

AJs are already established in the neuroepithelium of the neural tube, which is a precursor to the brain [45], and therefore genetic deletion of *afadin* starts at E10.5 after the establishment of AJs in the *afadin*-cKO mice. To examine whether AJs are maintained at the cerebral aqueduct and the third ventricle during development in the *afadin*-cKO mice, we immunostained afadin, nectin-1, and N-cadherin using their respective Abs. In the *afadin*-cKO brain at E13.5, the signals for afadin, nectin-1, and N-cadherin were faintly observed at the surface of the cerebral aqueduct but were not observed at the third ventricle as compared with those of the control brain (**Fig. 5** **, A and C**). At P0, the signals for afadin, nectin-1, and N-cadherin disappeared at the apical surface of the cerebral aqueduct and the third ventricle in the *afadin*-cKO brain (**Fig. 5** **, B and D**). These results indicate that the afadin protein was gradually reduced and finally disappeared at AJs in radial glial and ependymal cells at the cerebral aqueduct and the third ventricle of the *afadin*-cKO mice and that this gradual deletion of the afadin protein caused the destruction of AJs, leading to the disappearance of these cells from the cerebral aqueduct and the third ventricle, and stenosis of the cerebral aqueduct and obliteration of the third ventricle.

**Figure 6:**
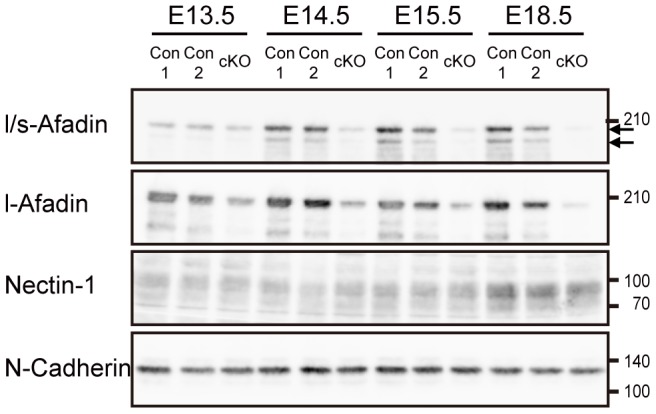
Gradual deletion of the afadin protein in the *afadin*-cKO brain. Lysates from the embryonic telencephalons were subjected to Western blotting using the indicated Abs. Arrows indicate the positions of l-afadin (upper) and s-afadin (lower) proteins. Molecular weight markers (kDa) are shown in the right. Con1, *afadin* ^+/f^; Con2, *afadin* ^+/f^; nestin-Cre; cKO, *afadin* ^f/f^; nestin-Cre.

In the last set of experiments, to confirm that the afadin protein was indeed deleted in the *afadin*-cKO brain and to examine whether the protein levels of nectin-1 and N-cadherin were changed or not, we performed Western blotting using their respective Abs and another anti-afadin Ab that recognizes both l-afadin and s-afadin (l/s-Afadin Ab). We used the telencephalon for this Western blotting, because it was practically difficult to obtain the mesencephalon containing the cerebral aqueduct and the third ventricle. Two protein bands with molecular masses of 205 and 190 kDa were detected by the l/s-afadin Ab in the telencephalon of the control embryos at E13.5, E14.5, E15.5, and E18.5 and only one band with a molecular mass of 205 kDa was detected by the anti-l-afadin Ab (**Fig. 6**). The intensities of both bands were slightly reduced in the *afadin*-cKO telencephalons at E13.5 as compared with those in the control telencephalons, but were further reduced at E14.5 and E15.5, and then were hardly detected at E18.5. Conversely, the protein levels of nectin-1 and N-cadherin were unchanged between E13.5 and E18.5, indicating that the disappeared immunofluorescence signals for nectin-1 and N-cadherin were not due to the reduction of these proteins. These results suggest that the afadin protein is gradually reduced and finally deleted without changes of the protein levels of nectin-1 and N-cadherin also in the mesencephalon.

## Discussion

To clarify the role of afadin in the development of the CNS, we generated the nestin-Cre-mediated cKO mice of the *afadin* gene and showed here that the genetic deletion of *afadin* in the brain caused hydrocephalus with stenosis of the cerebral aqueduct and obliteration of the ventral part of the third ventricle. In the *afadin*-cKO mice, stenosis of the cerebral aqueduct and obliteration of the third ventricle were observed at P0, but not at E13.5, indicating that the stenosis and obliteration gradually progressed during development. Hydrocephalus occurs by various mechanisms; excessive accumulation of CSF in the ventricles owing to an overproduction of CSF by the choroid plexus, defect of CSF reabsorption from the subarachnoid space, and impaired CSF flow [29]. We did not study here the effect of the genetic deletion of *afadin* on the overproduction of CSF by the choroid plexus and/or the defect of CSF reabsorption from the subarachnoid space. However, our results indicate that hydrocephalus in the *afadin*-cKO mice was initiated and progressed at least by impaired CSF flow owing to stenosis of the cerebral aqueduct and obliteration of the ventral part of the third ventricle, although a contribution of different mechanisms cannot be excluded for the hydrocephalus in the *afadin*-cKO mice.

Dilatation of ventricles and elevation of intraventricular pressure with hydrocephalus have harmful effects on the parenchyma and lead to edema, oxidative stress, proteolytic damages in the white matter, cell death, and reactive changes in glial cells [46]–[48]. In particular, gliosis contributes to the progression of the development of hydrocephalus by plugging the ventricle or the aqueduct. However, we did not detect reactive astrocytes at the stenosed area of the cerebral aqueduct and the obliterated area of the third ventricle at P0. It is unknown whether reactive astrocytes are observed in the earlier developmental stages, but the stenosis of the cerebral aqueduct and the obliterated area of the third ventricle induced by the genetic deletion of afadin is not accompanied with reactive astrocytes.

At the stenosed area of the cerebral aqueduct and the obliterated area of the third ventricle in the *afadin*-cKO mice at P0, we observed the fusion of the dorsal and ventral walls of the cerebral aqueduct and the bilateral walls of the third ventricles. This phenomenon was also found in the *hyh* mutant mice that developed hydrocephalus [31]. Conversely, in the physiological condition, tissue fusion processes are found during development of the palate, ventricular septum, neural tube, urethra, diaphragm, and eye [49]. Although they are anatomically different, the fusion mechanisms may be similar. When the fusion occurs, tissues proliferate and position themselves at a place close enough to fuse. Then, the epithelial cells of the apical luminal surface finally disappear or lose their epithelial features in various mechanisms, such as apoptosis, epithelial-mesenchymal transition, cell migration, denudation, or combinations of these mechanisms, depending on the organs [49]. Indeed, we observed that ependymal cells disappeared in the *afadin*-cKO mice, as shown in the *hyh* mutant mice [31]. The position of the dorso-ventral tissues of the caudal end of the aqueduct and bilateral tissues of the ventral part of the third ventricle are close enough to fuse, resulting in the stenosis and obliteration in the *afadin*-cKO mice. The tissue fusion mechanisms may be similar between the fusion shown here in the *afadin*-cKO mice and the physiological embryogenesis.

In this study, we showed that the denudation of ependymal cells and the aberrant location of radial glial cells were observed in the *afadin*-cKO midbrain. The denudation of ependymal cells has also been found in several mutants that display hydrocephalus [31], [34], [50], [51]. Conversely, the ectopic location of radial glial cells in the midbrain has not been described. During the development, radial glial cells, in addition to ependymal cells, also exist at the surface of the aqueduct and the ventricle, because radial glial cells initially proliferate or differentiate into neurons and then gradually differentiate into ependymal cells [42]. We found here that radial glial cells, which were identified as Sox2-positive cells, were scattered from the ventricular zone to the intermediate zone of the *afadin*-cKO midbrain. This phenomenon is explained by the detachment of the apical end feet of the radial processes from the surface of the third ventricle and cerebral aqueduct by the destruction of AJs in the midbrain, as observed in the cerebral cortex of the N-cadherin cKO mice [52]. The ectopic location of radial glial cells could not produce ependymal cells at the appropriate positions, the surface of the third ventricle and the cerebral aqueduct, causing the loss of ependymal cells at the surface. Consistently, we observed that the surface of the third ventricle and the cerebral aqueduct of the *afadin*-cKO midbrain was covered and replaced by neurons, but not by either radial glial cells or ependymal cells. These results indicate that afadin is essential for the maintenance of the position of radial glial cells, which allow them to produce ependymal cells, and the maintenance of the epithelial sheet structure of ependymal cells to prevent the denudation. Furthermore, the ectopic location of radial glial cells before their differentiation to ependymal cells would also cause the loss of ependymal cells.

Although the *hyh* mutant mice are alive after the birth and die by 2 months of age [53], the *afadin*-cKO mice died within 3 days after birth, indicating that the *afadin*-cKO mice developed more severe hydrocephalus than the *hyh* mutant mice by another mechanism in addition to the denudation of ependymal cells. The disappearance of radial glial cells from the surface of the third ventricle and the cerebral aqueduct may cause the impaired CSF flow during the development, accelerating the progression of hydrocephalus. It was shown that the ciliary defect in ependymal cells of the Celsr2/3- deficient mice developed hydrocephalus, owing to the impaired CSF circulation [54]. Radial glial cells as well as ependymal cells have primary cilia [55]. Primary cilia are well known as mechanosensory organelles [56]. During the developmental stage, radial glial primary cilia may function as detectors of the CSF flow to develop the planar cell polarity and to orient the flow, as shown in ependymal cilia [57], [58]. Thus, the combined mechanisms induced by the loss of radial glial cells from the surface of the third ventricle and the cerebral aqueduct, in addition to the denudation of the ependymal cells, could result in more severe hydrocephalus than the *hyh* mutant mice.

AJs are essential for maintaining the epithelial sheet structure that covers the luminal surface of tubes, tubules, and cavity of many organs. The epithelial sheet structure is maintained not only by AJs but also by other cell–cell junctions, such as TJs, desmosomes, and GAP junctions, and cell-matrix junctions, such as hemidesmosomes [4], [59]–[61]. Epithelial cells attach to the basement membrane through hemidesmosomes and disruption of this apparatus causes detachment of the epithelial sheet [62]. However, AJs are particularly important in the sense that they regulate these other junctions and destruction of AJs causes detachment of epithelial cells [63]. Afadin is a key molecule for the formation and maintenance of AJs in cooperation with nectins and cadherins [14]. In addition, it is important for the formation of tight junctions and desmosomes [64], [65]. Consistently, we showed here that afadin was colocalized with nectin-1 and N-cadherin in radial glial and ependymal cells in the cerebral aqueduct and the third ventricle and that the genetic deletion of *afadin* destructed AJs and caused the denudation of ependymal cells and ectopic location of radial glial cells from the surface of the third ventricle and the cerebral aqueduct. Because AJs in the midbrain were already established before *afadin* was genetically deleted in the *afadin*-cKO mice, these results indicate that afadin is essential for the maintenance of AJs in radial glial and ependymal cells covering the wall of the cerebral aqueduct and the third ventricle and that the destruction of AJs causes the denudation of ependymal cells and ectopic location of radial glial cells.

It was shown that the genetic deletion of other components of AJs, such as β-catenin and α-catenin, and the cell polarity protein aPKCλ, numb, and numbl destructed AJs in neuroepithelial and radial glial cells of the ventricles and develops hydrocephalus [34], [50], [51], [66], [67]. Although it was not known whether the genetic deletion of these molecules causes the stenosis or obliteration of the cerebral aqueduct or the third ventricle, it would cause the phenotypes similar to those observed in the genetic deletion of *afadin*. The present results together with these earlier observations make it evident that AJs in neuroepithelial cells, radial glial cells, and ependymal cells lining the ventricular and aqueductal walls are essential for maintaining the physical and normal functions of these cells and for preventing hydrocephalus.

## Materials and Methods

### Ethics Statement

This study was approved by the Institutional Animal Care and Use Committee of Kobe University (Permit Number: P130205) and carried out according to the Kobe University Animal Experimentation Regulation. All efforts were made to minimize suffering.

### Mice

The *afadin*-floxed mice [27] and nestin-Cre transgenic mice [28] were described previously. The homozygous and heterozygous mice carrying the *afadin* conditional allele is referred to as *afadin* ^f/f^ and *afadin* ^+/f^, respectively. To obtain the nestin-Cre-mediated cKO mice of the *afadin* gene, *afadin* ^f/f^ mice were mated with *afadin* ^+/f^ mice expressing Cre-recombinase under the control of the nestin promoter. All of the experiments were performed by using littermates. The morning after coitus and the day of birth were defined as E0.5 and P0, respectively.

### Abs

All of the following primary Abs were purchased from commercial sources: rabbit anti-l-afadin (Sigma, St. Louis, MO, USA), rabbit anti-l/s-afadin (Sigma, St. Louis, MO, USA), rat anti-nectin-1 (MBL, Nagoya, Japan), mouse anti-N-cadherin (clone 32, BD Biosciences, San Jose, CA, USA), rabbit anti-S100β (Abcam, Cambridge, MA, USA), mouse anti-class III β-tubulin (clone Tuj1, R&D systems, Minneapolis, MN, USA), and rabbit anti-Sox2 (Cell signaling, Minneapolis, MN, USA) Abs. Alexa-Fluor 488, 555, and 647-conjugated and Cy3-conjugated goat secondary Abs were purchased from Invitrogen (Carlsbad, CA, USA) and Millipore (Billerica, MA, USA), respectively. 4′,6-Diamidino-2-phenylindole (DAPI) was purchased from Nacalai Tesque (Kyoto, Japan).

### Immunostaining

Mouse brains were dissected out from embryos and fixed at 4°C for 2 h in 1% paraformaldehyde in phosphate buffer (PB) for staining with the anti-S100β, anti-Sox2, and anti-class III β-tubulin Abs and in 2% paraformaldehyde, 4% sucrose, and 1 mM sodium pyruvate in Hank's balanced salt solution with calcium and magnesium (Invitrogen, Carlsbad, CA, USA) and 10 mM HEPES (pH 7.3) for staining with the anti-l-afadin, anti-nectin-1, and anti-N-cadherin Abs. The fixed brain samples were cryoprotected in 30% sucrose in PB and embedded in OCT compound (Tissue Tek, Torrance, CA, USA). The sections at 12-µm thickness were mounted on glass slides. The sections were washed once for 10 min in PBS(-) containing 0.05% Tween 20 (PBST), then blocked at room temperature for 1 h in 5% normal goat serum and 0.2% Triton X100 in PBS(-), and incubated overnight at 4°C with primary Abs, which were diluted in the blocking reagent for staining with the anti-S100β, anti-Sox2, and anti-class III β-tubulin Abs or in a Can Get Signal immunoreaction enhancer solution B (Toyobo, Osaka, Japan) for staining with the anti-l-afadin, anti-nectin-1, and anti-N-cadherin Abs. After being washed in PBST three times for 5 min, the sections were incubated at room temperature for 1 h with secondary Abs, which were diluted in the blocking solution for staining with the anti-S100β, anti-Sox2, and anti-class III β-tubulin Abs or in a Can Get Signal immunoreaction enhancer solution B (Toyobo, Osaka, Japan) for staining with the anti-l-afadin, anti-nectin-1, and anti-N-cadherin Abs. After being washed in PBST three times for 5 min, the samples were mounted in a FluorSave reagent (Merck, Darmstadt, Germany) and observed with LSM700 or LSM510 META confocal laser scanning microscope (Carl Zeiss, Oberkochen, Germany).

### Histology

Mouse brains were dissected and fixed in 4% paraformaldehyde in PBS at 4°C for overnight. For Figure 1B, the adult brains were dehydrated in ethanol series and embedded in paraffin. The paraffin sections (4-5 µm) were stained with HE. For Figure 2, the embryonic brains were cryoprotected in 30% sucrose in PB and embedded in OCT compound (Tissue Tek, Torrance, CA, USA). The frozen sections (12 µm) were stained with HE.

### Western blotting

Telencephalons were dissected, put in tubes, frozen in liquid nitrogen, and stored at −80°C until use. The tissues were homogenized with a Teflon-glass homogenizer in 20 mM Tris-HCl (pH 7.5), 1 mM EDTA, 1 mM Na_3_VO_4_, 10 mM NaF, and 1 mM phenylmethylsulfonyl fluoride, 10 µg ml^–1^ leupeptin, 1.5 µg ml^–1^ aprotinin. Then, salt concentration was adjusted to 150 mM NaCl and 10% (wt vol^−1^) glycerol in addition to the above components. The homogenates were centrifuged at 800× *g* at 4°C for 5 min and the supernatants were collected. Protein concentrations were quantified with BCA protein assay (Pierce, Rockford, IL, USA). Protein lysates (10 µg of protein each) were subjected to SDS-PAGE, transferred onto PVDF membrane sheets, and blotted with Abs. Immunodetection was performed with Immobilon Western (Millipore, Billerica, MA, USA) and LAS-4000 luminescent image analyzer (Fujifilm, Tokyo, Japan).
